# A Mixed-Methods Evaluation of Parent-Assisted Children’s Friendship Training to Improve Social Skills and Friendship Quality in Children with Autism in Malaysia

**DOI:** 10.3390/ijerph18052566

**Published:** 2021-03-04

**Authors:** Sing Yee Ong, Samsilah Roslan, Nor Aniza Ahmad, Ahmad Fauzi Mohd Ayub, Chen Lee Ping, Zeinab Zaremohzzabieh, Seyedali Ahrari

**Affiliations:** 1 Faculty of Educational Studies, Universiti Putra Malaysia, Seri Kembangan 43400, Malaysia; syee718@gmail.com (S.Y.O.); nor_aniza@upm.edu.my (N.A.A.); afmy@upm.edu.my (A.F.M.A.); z_zienab@upm.edu.my (Z.Z.); seyedaliahrari@upm.edu.my (S.A.); 2 Department of Psychology, School of Medicine, International Medical University, Kuala Lumpur 57000, Malaysia; Nicole_Chen@imu.edu.my

**Keywords:** friendship skills, social skills, parent-assisted children’s friendship training, children with ASD

## Abstract

*Background*: This study evaluates the effectiveness of parent-assisted children’s friendship training intervention for enhancing friendship quality and social skills among children with autism spectrum disorders (ASD). We conducted a quasi-experimental study to investigate the effective outcomes of social skills and friendship quality in the pre-and post-parent-assisted CFT intervention phases; *Methods*: to conduct a 12-week field session, 30 children with their parents were selected. The Social Skills Improvement System Rating Scales and the Quality of Play Questionnaire-Parent were used to assess the effectiveness of the parent-assisted children’s friendship training during pre-and post-intervention. A semi-structured interview with parents was conducted at the end of the session; *Results*: findings revealed that intervention improved the social skills of these children. Additionally, the friendship quality of children with ASD improved before and after the intervention, however, engagement remained unchanged. Parents also showed some sort of improvement after the session as they reported a heightened sense of fear and resistance, awareness, learning and adjustment, change is not easy, and identifying support; *Conclusions*: there was clear evidence that children with ASD benefitted from parent-assisted CFTs in terms of social skills and friendship quality. However, larger and controlled studies are required to draw firm conclusions about this kind of intervention.

## 1. Introduction

Autism or autism spectrum disorder (ASD) is a condition that impacts a child’s development in two core areas: the first is social communication and social interaction, and the second is restricted, repetitive patterns of behavior and interests [1]. As the number of children with ASD is increasing globally, concerns about their neurocognitive and behavioral problems are rising [2,3]. Statistics indicated that at least one in 600 children in Malaysia has ASD [4]. Previous studies showed that children with ASD also exhibit considerable social skills deficits [5]. They have difficulties specifically in displaying social and emotional responses as well as reading silent social cues. It is observed that even high functioning children with ASD fail to develop and sustain relationships [6]. 

According to Rumney and MacMahon [7], the social skills deficits of these children significantly interfere with social relationships. This is apparent when they desire to engage with others but sense that they are socially limited stemming from having superficial friendships and lacking in social skills including social opportunities [8]. This can contribute to social failures, such as academic failure and school withdrawal, thus a significant area for early intervention [9]. Given the social skills deficits attributed to ASD and their effects and that long-term social adjustment for children is directly related to the development of appropriate social competency, interventions that address the social competency needs and concerns of individuals with ASD appear critical in overcoming many of the negative and debilitating effects of these disorders [10].

Among children, friendship is defined as a deep connection between peers in which they reciprocate and interact socially. Children with ASD tend to have a harder time developing friendships as a social skill because the understanding of friendship among them tended to focus more on companionship rather than intimacy or affection [11]. As such, perceptions of friendship quality between children with ASD differ from other children since the natural development and transmission of peer etiquette requires generally positive and sustained interaction with peers and learning from best friends [11]. Among children with ASD studied by Sigman [12], only 27% of them professed to have a best friend. Clinicians admittedly find it challenging to assist these highly driven children with impaired social skills and friendships. Besides, friendship-making programs for children with ASD tend to focus more on promoting rather than developing and training their social skills [13].

On top of that, the efficacy of such programs was reported as limited due to the possibility of omitting other possible factors that are important for these children. As the child with ASD matures overtime, continued isolation further magnifies their knowledge deficits in peer etiquette. Therefore, effective interventions must be suited to their unique needs.

### Parent-Assisted Friendship Training for Friendship Quality and Social Skills of Children with ASD

In previous programs for children with ASD, those who underwent social skills training have not achieved broad improvements in social adeptness that are meaningful and long-term [14]. Although numerous researches on children with ASD have pinpointed distinct cognitive and linguistic ineptness, there are insufficient studies that investigate the distinct deficits that arise from developing and maintaining friendships [15]. Besides, social function interventions have yet to be tested comprehensively in terms of their effectiveness in impacting peer acceptance and close friendship outcomes. According to Frankel and Myatt, [16], their manualized children’s friendship training (CFT) focuses on the aforementioned limitations. Its effectiveness has been validated after being field-tested on more than 1,000 children with ASD in numerous research and clinical environments. According to Ashman [17], understanding basic social rules of children with ASD is built by their parents’ specified instructions. Therefore, parent-assisted CFT can contain components that address social etiquette and specific behavioral rules, all of which are usable in peer-group settings, thus easing these children’s understanding of their social surroundings. CFT’s parent-structured and supervised playdates can address two areas: a child’s reputation within his/her circle of friends and the development of a close friend [18]. 

Parent-assisted CFT provides a valid ecology of service accessibility. Studies have reported parents’ resoluteness in learning evidence-based intervention strategies where they use it to improve their child’s adeptness in social settings [19,20,21]. Yet, other studies reported the absence of effect robustness on child outcomes [22,23]. Nevertheless, parents-assisted CFT offers benefits that cannot be denied, such as increased generalization and sustenance of a child’s skills [24], improved parents’ self-confidence [25], and reduced stress among parents [26]. Despite the aforementioned benefits, research has reported that less than 25% of parents have accessed these interventions in community settings for their ASD child [27]. Nevertheless, parents-assisted CFT can train their children with ASD to create ample interaction opportunities with friends of similar neurotypical age, thus establishing a social network for themselves [28]. Herein lies the importance to evaluate the effective role of parent-assisted CFT towards friendship quality including the network-building skills of children with ASD. Thus, the aim of this study was to test the effectiveness of CFT as an intervention to improve social skills and friendship among children with ASD.This study attempted to answer the following research questions:

RQ1: What are the roles of parent-assisted CFT for friendship quality?
RQ2: Does parents’ CFT affect the social skills of children with ASD?

## 2. Materials and Methods

### 2.1. Participants

Registered children with ASD and their parents volunteered for this study via online promotion in social networks. The criterion sampling was implemented to recruit suitable participants who shared similar characteristics based on the clinical criteria of the DSM-IV-TR [29] and the diagnostic criteria of the DSM-5 [30]. Parents provided their child’s diagnostic assessment report determined by the medical professionals in either private and government medical professionals including trained clinical psychologists and developmental pediatricians. The children were three girls and 27 boys as these were those registered voluntarily. None of them were on any medication, and they were not involved in any join non-pharmacological interventions when they participated in this intervention. Non-verbal IQ of the children ranged from 75 to 135 on the Raven’s Coloured Progressive Matrices Test [31].

Moreover, eight children (26.6%) were 7 years old and five children (16.66%) were 9 years old, respectively. Of the 20 children for which we obtained a full differential diagnosis, there were 17 children (56.67%) with a diagnosis of ASD while five children (16.67%) were diagnosed with ASD and Attention Deficit Hyperactivity Disorder (ADHD). Three children (10%) were diagnosed with Pervasive Developmental Disorder Not Otherwise Specified (PDD-NOS). One child (3.33%) was diagnosed with ASD and Attention Deficit Disorder (ADD) (see Table 1). The majority of the children (*n* = 28, 93.33%) came from a two-parent family and only two children (6.67%) came from a single-parent family (see Table 2).

### 2.2. Study Design 

An mixed methods design with a sequential approach was used to collect data. This method was chosen to combine both quantitative and qualitative approaches to create a design that contribute to the validity of the results through triangulation. This is in congruence with the ideas of the pragmatic worldview. In a pragmatic worldview both objective and subjective viewpoints are taken into consideration within the participant-researcher relationship [32]. For this study objective data were obtained through the questionnaire, and subjective viewpoints were obtained through the interviews in which the researcher was a coparticipant. We believes deeper insight into the parents’ perspective on the effects of CFT and their ability to manage their child’s social skills and friendship is very important. Thus, it is vital to understand and consequently create interventions and experiences that benefit both the child and the family.

In addition, the quantitative portion of this study used a quasi-experimental method of one-group pretest-posttest design. This type of design is appropriate because the study included an intervention (CFT) without randomization or a control group. One-group pretest-posttest design was adopted as it has widespread usage in applied field settings and can help enhance the internal validity of the study. Just because it is a generalized design in field studies does not mean that it is a design free of threats to validity. Internal validity such as maturation [33] may happen due to the CFT participants’ continued growth and learning in three months of the intervention. Qualitative data were obtained through semi-structured interviews with participants and observation of interventions. Field notes were used to complement the interview data. Field notes were taken while we observed the CFT or other experiences described by the families in the interviews. 

### 2.3. Variables and Hypotheses

The independent variable of the study is Children’s Friendship Training (CFT), where both parents and children were being taught of skills in the 12-session intervention Meanwhile, two dependent variables were included:social skills and friendship. 

The present study put forward the following hypotheses:

**Hypotheses** **(H1).** *There is a significant improvement in the social skills of children with ASD before and after exposure to CFT.*

**Hypotheses** **(H2).** *There is a significant improvement in the friendship quality of children with ASD before and after exposure to CFT.*

### 2.4. Intervention 

The participants underwent a weekly CFT intervention program of 90 minutes per session which ran for 12 consecutive weeks. The program was inspired by and adapted from a previously developed CFT program as part of the UCLA Children’s Friendship Program in 1991 [16]. This program focuses on key targeted skills such as social connection formation with the aid of parents, trading information with peers, peer entry into a group of children already at play, playdates, and conflict evasion and deliberation. The teaching methodology in building these skills is known as behavioral rehearsal intervention [34].

Twelfth sessions included helping the children with social situations such as making fun of a tease, “unjust” adult accusations, rules of good winners, and ways to stay out of a fight. For each session, four components were conducted: homework review, didactic, real play, and homework assignment. The graduation party was held during the final session.

The first session. The session began with the children talking about homework progress. Then, the core lesson was taught using the ‘Socratic Method’, which involved encouraging the children to create the rules for the activity. The researchers aimed to provide the opportunity for the children to be game creators to encourage active, livelier, and mutual learning with peers. When they contributed authentic ideas to the rules, they felt their sense of competency. Next, role plays were conducted for the children to practice the newly learned knowledge and skills with other participants. Throughout the session, tools for behavioral adjustment included stars and tokens for positive reinforcements while timeouts were used for punishments [35]. To wrap up the session, homework assignments were given to the children whereas handouts to outline session activities were given to parents. The first CFT parents’ session ensued, where goals were set and introduced to ensure every parent is aligned towards a unified, clear vision. Parents were also briefed on the limitations of the intervention to manage parents’ expectations of the program outcomes. 

The second session. This session required parents to practice their active listening skills. Discussions were had, specifically on the topic of how to be a good listener when their child shares about their day, the activities they had done, and their phone conversations with their peers, if any. The objective was to activate and stimulate two-way conversations between the parents and their child with ASD. 

The third session. This session required parents to gather resources for their children to facilitate forming new friendships. The aim was to assist their children in accomplishing their assignments related to improving their social skills in making friends. 

The fourth and fifth sessions. These sessions introduced the “slipping in” strategy where parents learned the tools which their children can use to make new friends. They were then required to practice the “slipping in” skills with their children. 

The sixth session. This session introduced the “inside games” topic where parents were asked to identify toys or indoor games that are suitable for their children’s playgroup. Parents were encouraged to assist their children in seeking out potential best friends. 

The seventh session. This session introduced the topic of playdates to parents. The main activities were discussions and agreeing on the right time for their children to call the parent of the child they wish to invite over for the playdates. 

The eighth session. This session briefed and guided parents on effective ways their children can overcome teasing by peers. Both parent and child practiced, among other ways, turning the tease into something light and funny. 

The ninth session. This session expanded from the eighth session to introduce appropriate ways to face unfair accusations by adults and respond accordingly. Both parent and child were given opportunities to practice this scenario. 

The tenth session. This session introduced parents to the topic of gender differences in play engagements including awareness of friendship patterns, the latter which parents need to adjust to their child’s anticipation of possible situations. 

The eleventh session. This session, built children’s skills which they can use to avoid and reduce physical conflicts with others. A graduation party was held at the end of this session. Follow-up assignments for the post-intervention period were explained to parents. Parents were reminded to ensure that their children continue to practice all the skills learned and the CFT fortification tasks. 

The twelfth session. This final session provided the platform for the children to practice their developed social skills in various real-life settings, such as a house, a school, and more. At this stage, the researchers postulated that the CFT program would have influenced social skills, problem behavior, and quality of play of the children with ASD. To achieve the goals, a pilot study was conducted in three phases: screening and selection, conducting the CFT sessions, and collecting pre-test and post-test data. 

### 2.5. Experimental Procedure 

This study used the quasi-experimental one-group pretest-posttest design. To do so, the researchers conducted an assessment (pre-test) before the intervention (CFT) began. After completing the intervention, an assessment (post-test) was conducted again. 

After the researchers received the parent and children’s consent during the consent session, the children were invited to another lecture room located in the Faculty of Educational Studies, Universiti Putra Malaysia (UPM). Here, they went through the mental status examination to make a reasonable estimate of the children’s communication and play skills. While waiting for their children to complete the mental status examination, parents were provided with an envelope that consisted of a biography form and assessment forms. The first assessment (pre-test) was completed when the parents completed the Parent’s Report. 

Both parents and children attended the CFT sessions concurrently. Each parent in treatment (CFT) was given a home assignment handout during CFT sessions [16] consisted of the guidelines and homework to be done weekly. Meanwhile, the children attended children’s sessions where they were taught skills in both indoor and outdoor activities [16]. Parents were taught how to provide support and monitor their children in friendship building. Children were taught different skills on how to: (a) communicate with others; (b) slip in; (c) handle rejection; (d) resist teasing; (e) respect adult supervisors; (f) practice good sportsmanship; (g) be a good host during a playdate session; (h) manage competition, and (i) avoid physical fights.

The second assessment (post-test) was done on the twelfth week of the treatment session. Parents were again being provided with an envelope of three assessment forms. The Child Report and Parent Report were collected after a week. 

The researchers together with a trainer who has relevant education qualification with at least a minimum of one year of working experience with special needs children administered the training jointly. 

The CFT sessions were conducted using two lecture rooms and an outdoor space, which fulfilled the following criteria as suggested by [16]:

(1) Parent room: The room should have a large table and enough space for all parents to be seated together at the same time.
(2) Child room: The room should have whiteboards, tables, and chairs for children to sit in.
(3) Play deck: The outside play area is used to teach skills for outdoor games and should resemble a schoolyard, as much as possible.

Therefore, the researchers investigated the effectiveness of parent-assisted CFT on friendship quality and the network-building skills among children with ASD through three phases: screening and selection, treatment, and assessment.

### 2.6. Measures

#### 2.6.1. Socio-Demographic Information

Basic demographic information was provided by the parents. In addition to the age and gender of their child with ASD, parents provided the age of the primary caregiver as well as their gender, education level, and marital status.

#### 2.6.2. Quantitative Evaluation of Program Perceptions

The Social Skills Improvement System Rating Scales (SSiS- RS) questionnaire was developed by Gresham and Elliot [36] in assessing social skills, problem behavior, and academic competence. To determine the social skill in CFT, the researcher only took 42 items in social skills in the parent’s form. Items in the questionnaire were rated as “Never”, “Seldom”, “Often”, and “Almost Always”. There are seven subscales in this instrument and they are cooperation, assertion, responsibility, self-control, empathy, engagement, and self control [16]. It was reported that the authors developed SSiS- RS using the content from literature searches and item selection by clinicians, parents, and other education professionals. SSiS-RS showed a moderate correlation with Behaviour Assessment System for Children (2nd Ed.) and the Vineland Adaptive Behaviour Scales (2nd Ed.) [36]. Based on the result of the pilot test, the scale reliability coefficients were 0.883 for the parent’s form. 

The Quality of Play Questionnaire-Parent (QPQ) was developed by Frankel and Mintz [37]. The researchers employed a 19-item questionnaire to measure the frequency and children’s quality of play during their recent playdates. It had three factor-based scales to measure the children’s quality of play, namely, conflict, engage, and disengage scales. The participants responded to a 4-point Likert scale, ranging from 0 (Not at all), 1 (Just a little), 2 (Pretty much), and 3 (Very much). There were two open-ended items: (i) item 18 required the parents to recall and report the frequency of their children being invited to play at another child’s house as the only guest in the last month, and (ii) item 19 required the parents to recall and report the frequency of their children inviting another child to their house as the only guest to play in the last month [16]. The result of the test indicated a reliability coefficient of 0.750 for the Conflict factor-based scale, 0.698 for the ‘Engage’ factor-based scale, and 0.736 for the ‘Disengage’ factor-based scale.

### 2.7. Qualitative Data

A semi-structured interview technique was employed to collect qualitative data [38]. The preference for this approach was higher for two reasons; first, it provides a structure that accommodates specific ideas and concepts that are related to the research topic for discussion, and second, it provides sufficient flexibility to facilitate emergent ideas that can be explored further if they were relevant to the research topic. By using this approach, the researchers could better understand the process of skill development and the subsequent impact of the strategies being introduced. Extensively trained psychologists, all participating in regular booster sessions to avoid interview drift, administered the structured diagnostic interviews (SCID-5: [39]). Three-trained mental health professionals who were familiar with the DSM-5 classification and diagnostic criteria administered it.

### 2.8. Ethical Aspect

The Ethics Committee of the Ministry of Health of Malaysia (NMRR ID: 17-321-343530, IIR) and the UPM ethical committee (JKEUPM: FPP 040; 2017) have examined the ethical considerations of this human-subjects study. All ethical guidelines adhered to the highest standards. The ethics approval was granted based on the following fulfillment: (i) good clinical practice, (ii) confidentiality of the information, and (iii) prior informed consent.

### 2.9. Protection of Human Subjects

The researcher also obtained written consent from parents and children (in witness of their parents and teachers) participate in CFT before they started the session. All data obtained in this study are kept and handled confidentially, following applicable laws and/or regulations. The study subject’s participation in this study is voluntary. After completion of this study, the researcher had obtained approval from UPM and the Director-General of the Ministry of Health (MOH) before publication. 

### 2.10. Data Collection

**Figure 1:**
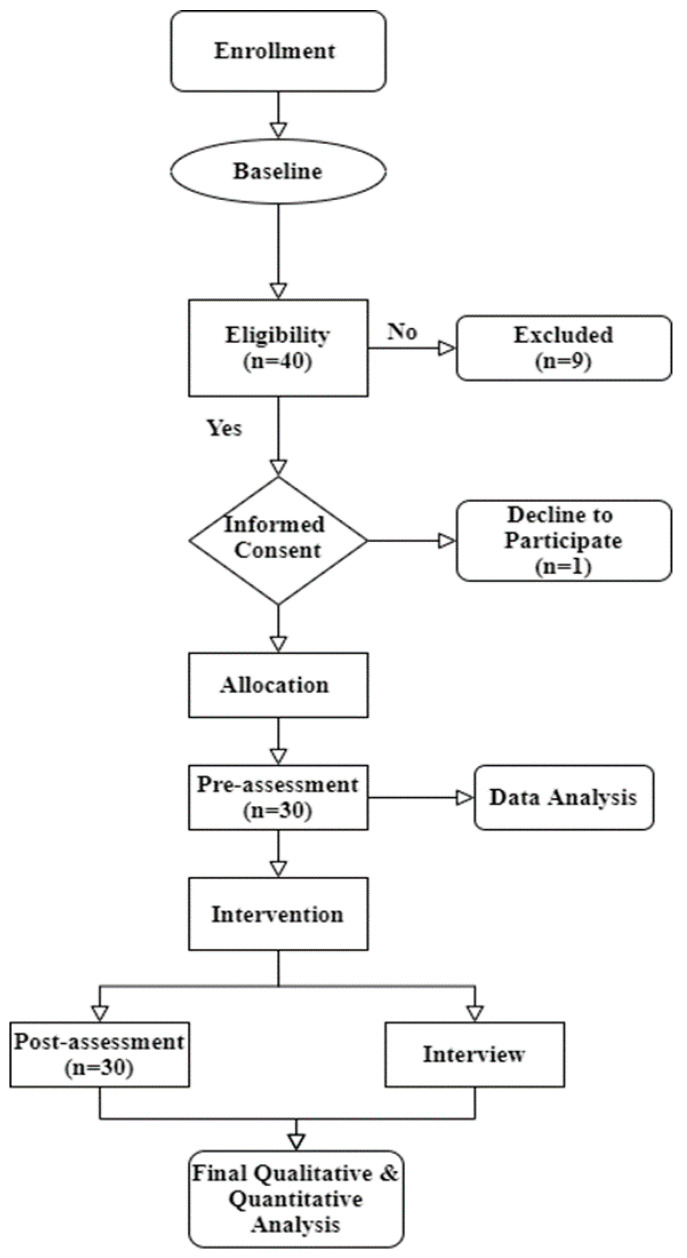
Research flowchart of the study.

Table 3 presents the data collection schedule. The baseline assessment measures the respondents’ competencies. All T1 and T2 data were collected within 12 weeks. The efficacy endpoint was measured at follow-ups in weeks 4 and 12, respectively (see Figure 1). 

### 2.11. Data analysis 

To analyze the data collected for this study, Version 24 of the Statistical Package for the Social Sciences (SPSS; IBM Corporation, Armonk, NY, USA) was employed to generate descriptive statistics and inferential statistics for interpretation. The transcript qualitative data obtained from the interviews were analyzed by thematic analysis [40] by two independent researchers.

## 3. Results

### 3.1. Descriptive Analysis

There were 27 (90%) male children participants and three (10%) female children participants in the CFT program. The majority of the children were 8 years old (*n* = 10, 33.33%) when they participated in the CFT program. There were 19 (63.33%) children who professed to have no friends at school, 14 (46.67%) children were facing classroom behavior problems, and 12 children (40%) were being teased. Besides, 12 children (40%) had no one seeking him/her out at school, and three (10%) were aggressive to peers. Before participating in the CFT, the majority (*n* = 18, 60%) of the children had no playdates while 12 children (40%) had less than two playdates per month, which is categorized as infrequent playdates. When the children played with their friends, 16 of them (53.3%) were disagreeing occasionally, 11 of them were generally playing harmoniously, and only three (10%) were bossy or frequently disagreeing with their playmates. In terms of their play skill, 24 of them (80%) said that they knew how to play basic board games, 23 of them (76.67%) wanted to have friends, and 13 of them (43.33%) knew how to play school recess games. There were 24 mothers (80%) and six fathers (20%) who participated in the CFT. 

### 3.2. Results of Quantitative Data

Analysis of the kurtosis, skewness, and equality of variance of key measures indicated that the data met the assumptions underlying parametric tests in terms of normality of distribution and equality of variance across the sample. Paired-samples t-tests were conducted to evaluate the effect of the parent-assisted CFT intervention on children’s social skills and friendship quality. Means, standard deviations, and t-statistics are shown in Table 4. Table 4 shows statistically significant differences in pre- to post-intervention scores for social skills (t (29) = −2.7, *p* = 0.011), friendship–disengage (t (29) = 4.974, *p* = 0.000), and friendship–conflict (t (29) = 4.328, *p* = 0.000). However, friendship–engage remained unchanged. 

Besides, Cohen’s d indicates a major effect (Cohen’s d = 1.000). These results suggest that based on parents’ evaluation, CFT leads to significant social skills improvement among children with ASD. Thus, Cohen’s d indicates a large effect of friendship– disengagement (Cohen’s d = 1.2114) based on parents-assisted CFT and does not significantly improve the engagement behavior of children with ASD. The results also demonstrate a large effect size for the friendship–engage (Cohen’s d = 0.9881), the large effect size for the friendship–conflict (Cohen’s d = 0.9936), post-participation in the CFT group intervention.

### 3.3. Results of Semi-Structured Interviews

This study used Braun and Clarke’s [41] six-stage thematic analysis process to analyze the semi-structured interview transcripts. In collaboration with the second author who is an experienced educational psychologist and researcher, the researchers developed the themes from interviews with parents (see Table 5). Following this, interviews were transcribed, and the texts were analyzed. The researchers highlighted the quotes from the text and transferred them onto a spreadsheet to identify themes and sub-themes. Cumulatively, process completion required five meetings that lasted one hour each. Table 3 presents the themes that emerged from the parent interview analysis.

For most parents, they fear their child making mistakes, and this was described by three parents as shared during the interview as “*I make sure that he doesn’t embarrass, like he doesn’t make mistakes like, too rough, not playing together or they run away*”. Another parent talked about the fear that his son did not meet her expectation which might bring embarrassment to her, as expressed by “*I avoid him playing with strangers. Because I don’t want any problem to occur*”. The second theme that emerged was related to parents’ awareness, learning, and adjustment during the 12 CFT sessions. Parents realized that they had the mindset of treating their children’s condition(s) as ’special children’ and need to be given more attention towards their ’special traits’ which limited their interaction with others, as expressed by “*We are now more aware the need to have a peer group for interaction all that*”. Two parents admitted that they have not been emphasizing their children’s development in social skills, outdoor play, as well as two-way communication, as expressed by “*We realized that the playdate is important to point must they get*”. During the CFT sessions, all parents acknowledge that they learned many things (“*I also learned. So, not just to bring, let him join … not only him, I learn a lot more, to know how to help him*”). There was a process of adjustment for the parents in CFT as well, and they all described their efforts in adjusting to CFT as “*Even after this session, I just Whatsapping … we need to create a more outside school on peer time*”. The third theme that was observed was that change is not easy. Parents went through several challenges when they were attempting to make calls to other children, practicing “slipping in” exercises in group play, and inviting other children for a playdate session. Five parents stated that the relationship with the invited potential guests had determined the invitation (out-group call, playdate session) acceptance, as expressed by “*We can’t invite the neighbor kids to house, to the house to play date because we don’t know their parents*”. Finally, it was noticeable that parents sought help from people they knew within their circle, engaged in weekly group discussions, had self-initiative, and sought professionals to jointly conduct the practices with their children (“*…Then, playdates sometimes, I got to invite the special child. Then, I know the other parents and all that, so I got to highlight that this kind of things very important…*”).

## 4. Discussion

This study is the first to evaluate the effectiveness of a parent-assisted CFT intervention which is adapted for children with ASD. This novel study provides significant findings. Outside of Malaysia, CFT was already explored by researchers as an intervention in friendship formation, but it has not been used and adapted specifically for children with ASD in the capital of Malaysia, Kuala Lumpur. This study aimed to depict the suitability of using parent-assisted CFT for children with ASD in Kuala Lumpur by comparing the pre-test and post-test scores in friendship quality social skills. The parents conducted the outcome measures of their children with ASD who participated in this study, and they presented findings that indicated some evidence of significant social skills enhancement of their children with ASD after CFT completion. This is consistent with the findings by Frankel [42], who extended the parent-assisted CFT literature for children with ASD, and subsequent research that focused on long-term outcomes of parent-assisted intervention that addresses the social skills of high-functioning children with ASD [43].

As noted, these children’s social skills did improve in this study, and one plausible explanation was because, throughout the CFT program, they picked up social cues, such as the right place and the right time to make friends. This reflects the finding of previous studies that reported children with ASD fail to make friends because they lack specific knowledge and concepts of making friends [e.g., 44]. Once sufficient knowledge on making friends are acquired, these children advanced to the second CFT session and beyond where they learn and practice having a two-way conversation with peers [44]. Another reason is that CFT is known for its unique intervention point which is called the “slipping in” activity. Here, the children with ASD could learn and practice the strategies in seeking their peers’ permission to indicate their wish to participate in a group play. This activity was found to be effective in empowering the ability of children with ASD to make new friends through better play quality. 

In this study, it is interesting to note that the result from parents’ evaluation indicated a decrease in the friend-disengage and friend-conflict behavior during playdates, which is not a significant difference in their engagement behavior at those activities. In other words, the results indicated that parent-assisted CFT is significantly effective in reducing friend-disengage and friend-conflict behaviors during play among children with ASD in Kula Lumpur. These results agree relatively well with Frankel [42], in that children who participated in CFT displayed lower conflict and disengage scores compared to children who received delayed group treatment. Furthermore, earlier research found that having a good quality friendship can significantly enhance global self-worth. Therefore, in the context of this study, quality friendships can help children with ASD enjoy higher self-worth when they are hanging out with peers under their parents’ guidance [45]. 

In terms of parents’ experiences, the findings indicated diverse experiences that may impact CFT programs. Four main themes were identified during analysis namely (i) fear and resistance, (ii) awareness, learning, and adjustment, (iii) change is not easy, and (iv) identifying support. The emerged themes echoed Carnall’s [46] Coping Cycle Model with a similar five-phase coping style which includes denial, defense, discarding, adaptation, and internalization. In this study, parents faced different challenges in the process of adjusting and developing new skills, which impacted the effectiveness of the parent-assisted CFT. Both parents and children faced time constraints to commit to the CFT as they always have a packed schedule. This challenge of the time constraint is supported by Teo and Lau’s [47] study and Moroz’s [48] research. It is an issue commonly faced by parents with special needs children in Malaysia and the USA. Tully [49] reported that time constraint is one of the barriers to participation among fathers (*n* = 1001) in early childhood intervention in the USA. Meanwhile, in Piškur’s [50] study, the lack of time was also a challenge that parents faced in supporting their special needs children’s participation in general physical settings.

Moreover, relationship closeness is highlighted among parents as an influencing factor in the success of CFTs as it can facilitate guests’ willingness to participate. In their opinion, the close relationship between both parents and children would help smooth out the playdate sessions for them to complete the weekly practice. This speaks to the collectivist culture in Malaysia [51]. They consider themselves as an important member of an inclusive and interrelated whole in the domains of their family, their workplace, their country, or their religious community [52]. They are more comfortable socializing within their in-group or with people whom they already knew [52]. This is a plausible explanation of why some CFT parents find it difficult to approach other parents for a playdate appointment. Besides, CFT parents had described being distracted from their CFT focus due to the multiple roles they have to play. One of the important traits of individuals in a collectivist culture is that their action is determined by norms, roles, and goals of their collective [52]. This describes the triple burden on CFT parents who need to juggle their roles as a spouse, a parent, and a child in their daily routine. They need to fulfill their tasks and responsibilities. 

It is also noticeable that their weekly CFT homework is impeded by children’s packed schedule of after-school activities and the emphasis on academic-related activities. Parents’ emphasis on the academic performance of their children reflects the collectivist culture, where children’s ability to compete with others is often highlighted [47]. This speaks to the different value practices in the macro-system of the family members that impact the special needs children’s functioning in interventions as proposed in the Bronfenbrenner Ecological Framework [53].

The ways CFT parents seek support were supported by Piškur’s findings [50]. In their study of 14 reviews, they concluded that parents engaged in four strategies, which include “networking”, “educating”, “advocating”, and “creating opportunities” when they were supporting their children’s participation in both school and home settings. The parents in CFT initiated to engage others to start the playdate sessions, created awareness among others, and built a relationship with the community, consisting of teachers, family members, and other parents, to get the homework assignment done through “networking”, “educating”, and “advocating”. This is consistent with the interview outcome of 11 parents of children with ASD [53], where these parents created awareness and advocated in managing their barriers. Next, parents in CFT adjusted to create opportunities for their children in their practices as well (“creating opportunities”). Parents in CFT seek help from their informal support system (other known parents and family members), and they also seek assistance from their functional and formal support system (CFT facilitators, and school). They expressed their appreciation towards the CFT parent group discussion which provided them alternatives to complete the CFT practices. This is similar to what the parents shared in Hall and Graff’s [54] research stating that the support group does help in information sharing as well as inspirational support. It is also important that they need to learn from the experts so that they could apply the skills with their children in the sessions. This is in tandem with the resulting outcome which states that parents tend to seek advice from a knowledgeable practitioner in managing their children [54]. 

## 5. Conclusions

The findings from this study presented empirical evidence on CFT’s effectiveness in enhancing social skills, disengagement, and conflict behavior in friendship. CFT does not show a significant difference in engagement behavior in friendship among children with ASD in Kuala Lumpur as an evidence-based intervention to address deficits of children with ASD in social competence. Therefore, continuous effort in assisting these families is needed in this country.

### 5.1. Implications of the Study

The researcher has carried out parent-assisted CFT as an intervention strategy that addresses social skills and friendship quality among children with ASD. Accordingly, the first major practical contribution of the present research is that it provides much needed empirical data on the effectiveness of parent-assisted CFT. The information provided in the current study shall allow the policymakers, interventionists, educators, and parents to design initiatives, tools, and actions based on what is being proposed in CFT to suit the Malaysian context. This is to address the needs of local interventionists who are currently solely relying on the empirical data in western countries’ context despite distinct cultural factors that need to be considered. According to the findings, parent-assisted CFTs can be introduced in primary schools and special needs centers to facilitate play engagements and peer interactions between children with special needs and others. This will help to address the difficult situations they are facing in schools, such as being bullied and being alienated by their regular classmates, or in the community. A final implication that stems from the research is the inclusion of parents in the intervention by empowering them with strategies to support their children with ASD daily because, in the majority common practice of intervention, interventionists hardly involve parents in the sessions. Whereas, parent-assisted CFTs provide participatory methods for the parents where they learn alongside their peers as they share alternatives and different perspectives in viewing their children’s issues in making friends. 

### 5.2. Study Limitations

This research offers encouraging insights into the potential effectiveness of parent-assisted CFT programs for children with ASD, and how it can change and support the development of their friendship quality and social skills. There are some limitations to the current study. First, the generalizability of this study is limited merely to children with ASD within Greater Kuala Lumpur. Thus, the selected sample may not be representative of children with ASD in other parts of Malaysia. As the duration of the treatment was 12 weeks, the factors that may contribute to changes towards the participants’ affection and mental state may be beyond control throughout the session. Hence, maturation may happen and if it culminates, this may influence the result of the group sessions. Finally, the children in this study were overwhelmingly male. Future studies could gather qualitative, as well as quantitative data, to examine the effect of parent-assisted CFT intervention on females with ASD.

## Figures and Tables

**Table 1:** The background characteristics of included children (*n* = 30).

	N (%)		N (%)		N (%)		N (%)
**Age**		**Diagnosis**		**Pragmatic Language IMPAIRMENT**		**Social Communication DISORDER ^1^**	
7	8 (26.6)	ASD	17 (56.66)	Receptive language disorder		Difficulties in Interpersonal communication ^2^	
8	10 (33.33)	ASD, ADD	1 (3.33)	Mild	0	Mild	2 (6.6)
9	5 (16.66)	ASD, ADHD	1 (3.36)	Moderate	18 (60)	Moderate	27 (90)
10	2 (6.66)	Asperger Syndrome	1 (3.36)	Severe	12 (40)	Severe	1 (3.4)
11	2 (6.66)	Asperger Syndrome, ADHD	1 (3.36)	Expressive language disorder		Difficulties in social initiation and response	
12	3 (10)	GDD	1 (3.36)	Mild	2 (6.66)	Mild	16 (53.34)
**School Type**		GDD, ADHD	3 (10)	Moderate	17 (56.66)	Moderate	12 (40)
Government School (Sekolah Harian)	13 (43.4)	PDD- NOS	5 (16.66)	Severe	11 (36.66)	Severe	2 (6.66)
Government School (PPKI)	6 (20)	**Degree of Autism**		Written expression disorder		Social interaction skills deficits	
Home School Center	6 (20)	Mild	1 (3.3)	Mild	6 (20)	Mild	2 (6.66)
International Primary School	3 (10)	Moderate	27 (90)	Moderate	20 (66.6)	Moderate	23 (76.66)
Private Primary School	2 (6.6)	Severe	2 (6.6)	Severe	4 (13.4)	Severe	5 (16.66)

Note; ASD = Autism spectrum disorder; ADD = Attention deficit disorder; ADHD = Attention deficit hyperactivity disorder; GDD = global developmental delay; PDD-NOS = Pervasive developmental disorder—Not Otherwise Specified. ^1^; Social communication disorder (SCD) is a diagnosis listed under DSM-5. ^2^; Interpersonal communication includes verbal and non-verbal communication.

**Table 2:** Background information of parents (*n* = 30).

	N (%)		N (%)
**Parents Race/Ethnicity**		SPM	3 (10.00)
Malay	7 (23.33)	**Parent Occupation**	
Chinese	17 (56.67)	Professional	16 (53.33)
Indian	6 (20)	Business	3 (10)
**Parent Religion**		Housemaker	11 (36.67)
Muslim	7 (23.33)	**Parents’ Marital Status**	
Buddhism	14 (46.67)	Married—living together	28 (93.33)
Christian	4 (13.33)	Single—divorced—separated	2 (6.67)
Hindu	5 (16.67)	**Parents’ Income**	
**Parents’ Education Level**		Low (less than RM4360)	11 (36.67)
Postgraduate	7 (23.33)	Medium (RM 4360-RM9618)	11 (36.67)
Degree	16 (53.33)	High (More than RM 9618)	8 (26.67)
Diploma	4 (13.33)		

**Table 3:** Schedule of the assessments.

Timepoint	Enrollment	Baseline	CFT Intervention			
	−1	0	Within 12 Weeks Follow up			
**Enrolment**						
Eligibility screen	✖					
Informed consent	✖					
Allocation						
**Interventions**			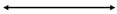			
**Assessment**						
Demographic	✖	✖				
**SSiS-RS**		✖				✖
QPQ		✖				✖
Interview						✖

✖ shows that activity took place in certain weeks; Double-headed arrow: The intervention’s length.

**Table 4:** Descriptive statistics and t-statistics.

Construct	Pre-Intervention		Post-Intervention		t	df	*p*-Value	Cohen’s d
	Mean	*SD*	Mean	*SD*				
Social Skill	70.70	12.804	79.40	4.97	−2.7	29	0.011 ^a^	1.000
Quality Friendship								
Friendship—Engage	5.53	2.776	5.60	2.238	−0.138	29	0.891	0.98
Friendship—Disengage	5.33	2.279	2.97	2.810	4.974	29	0.000 ^b^	1.21
Friendship—Conflict	9.57	5.302	4.93	4.799	4.328	29	0.000 ^b^	0.99

Note. ^a^ significant at alpha = 0.05; ^b^ significant at alpha = 0.001.

**Table 5:** Themes and subthemes from thematic analysis of parents’ interviews.

Theme	Sub-Theme
Fear and resistance	Fear of making mistakes Fear of their child creating behavioral issues with their peers Fear just to bring her child out Fear that their children do not meet their expectations Fear of interacting with other children
Awareness, learning, and adjustment	Awareness of the need to have a peer group for interaction Awareness of the need to help her child create a social network of friends Learning many things from CFT Learning to be assertive in teaching and guiding their children Realizing their efforts in adjusting to CFT
Change is not easy	Identifying time commitment as an utmost challenge for them Realizing the frustration of being rejected by the invited parents Realizing the lack of resources to get their homework assignment
Identifying support	Identifying support to complete their homework after the weekly session Seeking help from people they knew in their environment Initiating in promoting the importance of making friends
